# Gastrointestinal Bleed from Erosive Gastritis and Duodenitis: A Sentinel Event of Invasive Lobular Carcinoma of the Breast and a Diagnostic Dilemma

**DOI:** 10.7759/cureus.2757

**Published:** 2018-06-07

**Authors:** Mark Bilinyi Ulanja, Mohamed Taha, Arshad Al-Mashhadani, Bryce D Beutler, Marwah Al-Tekreeti, Christie Elliot, Santhosh Ambika

**Affiliations:** 1 Internal Medicine, University of Nevada School of Medicine, Reno, USA; 2 Internal Medicine, University of Nevada, Reno, School of Medicine, Reno, USA; 3 Internal Medicine, University of Nevada Reno, School of Medicine, Reno, USA; 4 Internal Medicine, University of Nevada, Reno School of Medicine, Reno, USA; 5 Public Health, 2) American Public University System, Charles Town, USA; 6 Pathology, University of Nevada Reno, School of Medicine, Reno, USA; 7 Hematology-Oncology, University of Nevada Reno, School of Medicine, Reno, USA

**Keywords:** invasive lobular carcinoma, breast, gastritis, duodenitis, signet ring carcinoma, cdk inhibitor, anemia, immunohistochemistry, gastrointestinal bleed, non-steroidal anti-inflammatory drugs (nsaid)

## Abstract

Metastasis from breast cancer to the gastrointestinal (GI) tract is uncommon, and such events presenting as GI bleeding are exceedingly rare. In some individuals, the absence of classical findings of primary breast cancer coupled with the non-specific nature of GI symptoms may make early detection and diagnosis challenging. Our patient is a 75-year-old female who presented with symptomatic anemia manifesting as progressive dizziness, weakness, and early satiety that developed eight days after right knee arthroplasty. She had a remote history of acid reflux disease and reported regular use of non-steroidal anti-inflammatory drugs (NSAIDs). Physical examination was notable for pallor and tachycardia; the cardiopulmonary examination was otherwise unremarkable and the abdominal examination was normal. A fecal occult blood test was positive. Subsequent esophagogastroduodenoscopy demonstrated significant erosive gastritis and duodenitis that was initially attributed to the patient's NSAID use. However, biopsy showed signet ring carcinoma. No gastric primary tumor was identified on work up. Extensive evaluation ultimately revealed invasive lobular carcinoma of the breast. Notably, no primary breast lesion had been detected on physical examination or breast mammography or magnetic resonance imaging (MRI). Therapy for invasive lobular carcinoma of the breast is substantially different from gastric carcinoma and thus it is important to accurately diagnose the condition early in its course to optimize patient outcomes.

## Introduction

Metastasis from breast cancer most commonly involves the bones, lungs, and/or the liver [1-2]. Metastasis to the gastrointestinal (GI) tract from breast cancer is uncommon, and such events initially presenting as GI bleeding are seldom described in the medical literature. When the GI tract is involved, the stomach is the most frequently affected organ. The vague constellation of GI symptoms coupled with the non-specific infiltrative pattern of metastatic lesions makes early diagnosis challenging, particularly in the absence of significant findings on breast imaging [3-4].

We present an unusual case of metastatic lobular carcinoma of the breast that was discovered during a workup for a presumed primary gastric cancer, posing a diagnostic dilemma.

## Case presentation

A 75-year-old Caucasian female presented to our emergency department (ED) with a one-week history of progressive dizziness, weakness, early satiety, and chest heaviness. Symptoms started eight days after right knee arthroplasty. Review of systems was negative for abdominal pain, nausea, vomiting, hematochezia, melena, and bone pain. Pertinent past medical history included a remote history of acid reflux disease, history of colonic diverticular disease, and regular use of nonsteroidal anti-inflammatory drugs (NSAIDS).

**Figure 1:**
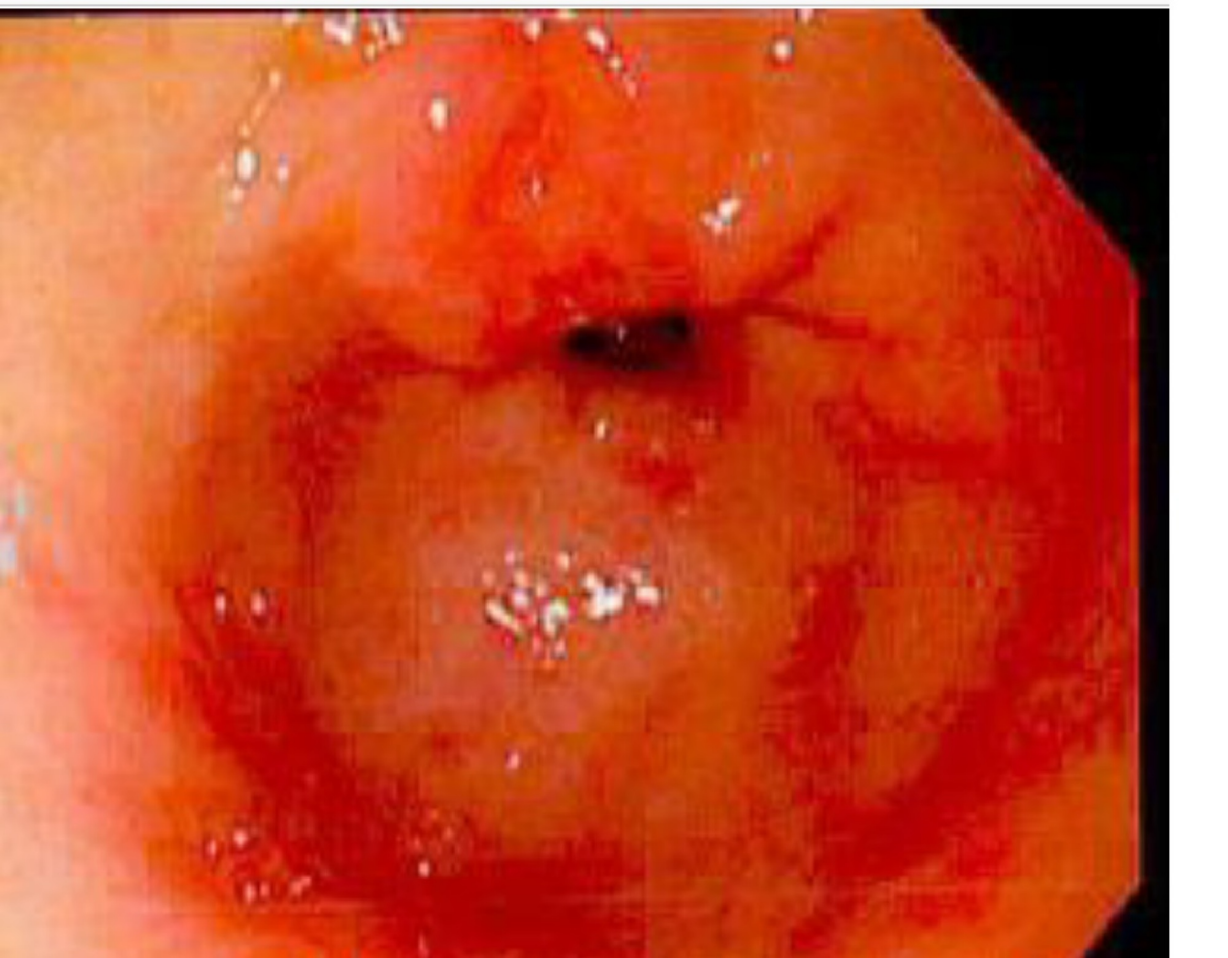
Esophagogastroduodenoscopy (EGD) demonstrating erosive gastritis

**Figure 2:**
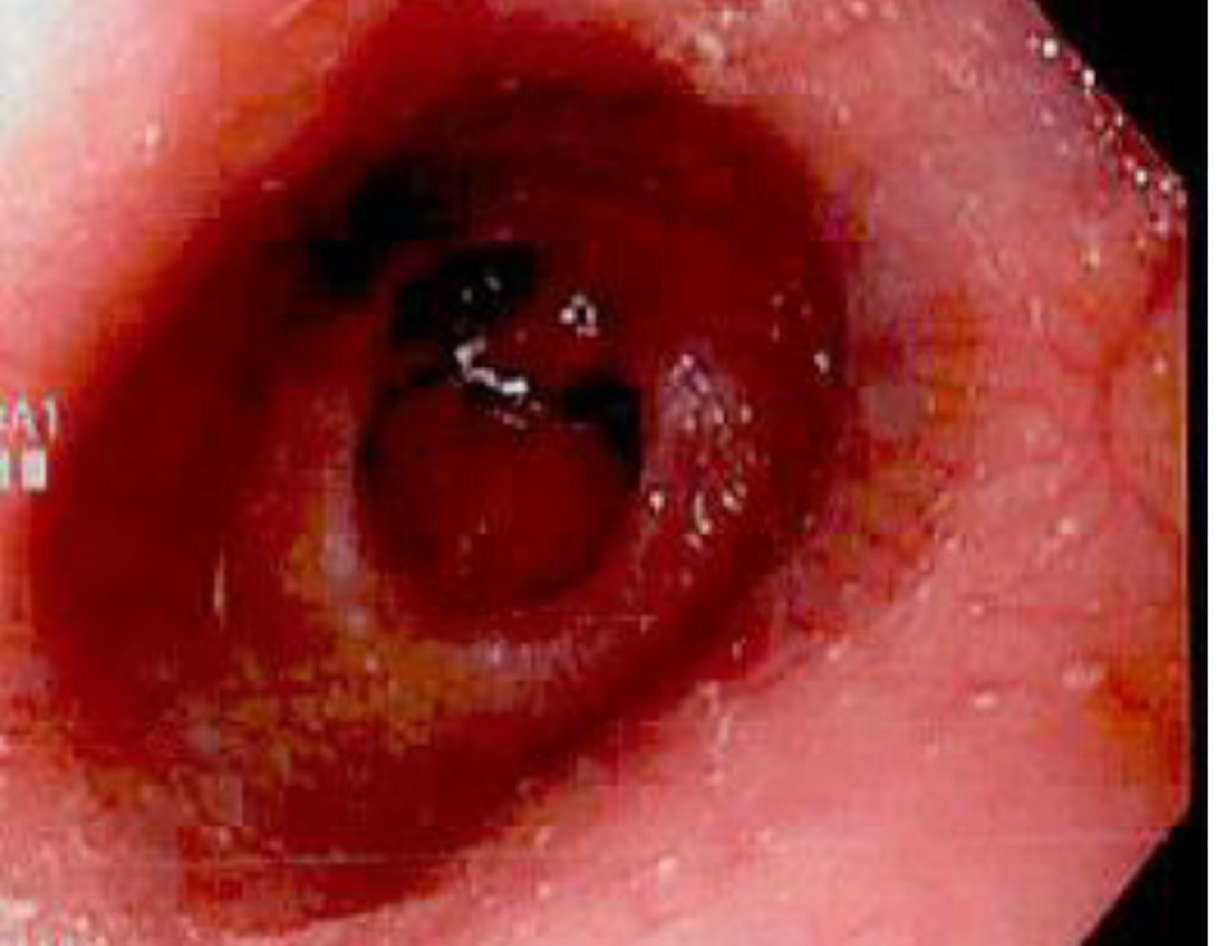
Esophagogastroduodenoscopy (EGD) demonstrating erosive duodenitis

Physical examination was notable for pallor, tachycardia, and positive fecal occult blood. Examination of the heart, lungs, and abdomen were unremarkable. Examination of the breasts performed in the ED was normal. Laboratory testing revealed a hemoglobin count of 7.6 g/dL, prompting further evaluation for a GI source for the bleeding. Esophagogastroduodenoscopy (EGD) revealed erosive gastritis and duodenitis as well as a non-obstructing Schatzki ring at the gastroesophageal (GE) junction (Figures 1-2).

The pathological evaluation of the gastric and duodenal samples was positive for signet ring carcinoma, favoring a presumptive diagnosis of linitus plastica (Figures 4-5). However, immunohistochemical (IHC) staining of the biopsy revealed positive estrogen receptor/progesterone receptor (ER/PR), GATA3, and mammaglobin with negative human epidermal growth factor receptor 2 (HER2)/neu and a negative E-cadherin (Figures 6-7).

**Figure 3:**
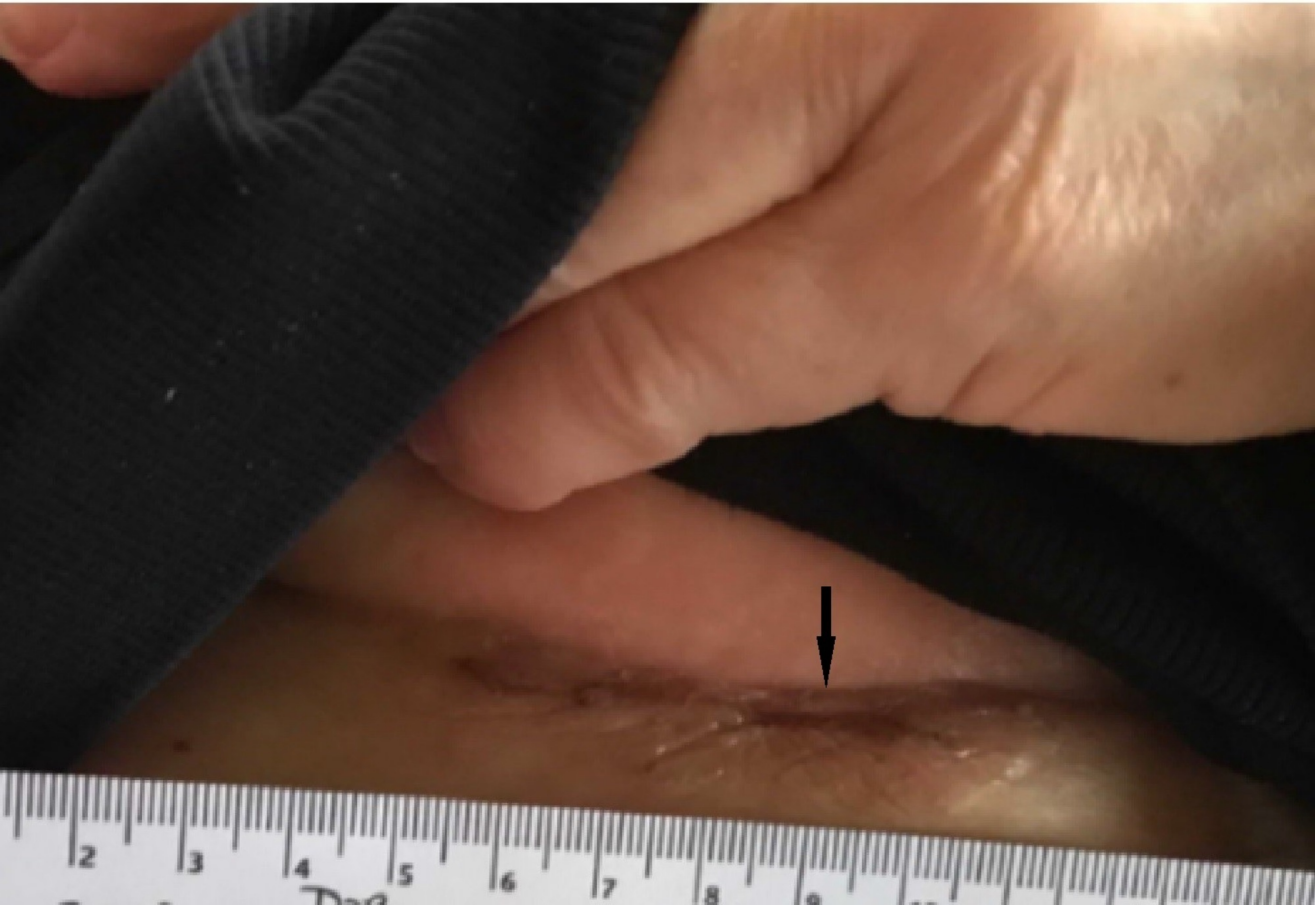
Inframammary skin thickening and induration

The above findings prompted further investigation for a primary source with high suspicion for the breasts. Detailed examination of the breasts revealed bilateral chest wall nodules concentrated in the inframammary area (Figure 3). Notably, the patient had obtained annual mammograms from 2009 to 2017, all of which were normal. Furthermore, magnetic resonance imaging (MRI) of the breasts failed to detect any abnormalities in the breast tissue. Computed tomography (CT) imaging of the chest, abdomen, and pelvis was remarkable only for mild mediastinal lymphadenopathy, an unsuspicious 5 mm pulmonary nodule in the right lower lobe, and hepatomegaly with cirrhotic changes.

Further workup revealed an elevated alkaline phosphatase of 188 U/L (normal range: 30-99 U/L). Liver and kidney functions were otherwise normal. Positron emission tomography (PET) scan was remarkable only for mildly increased uptake in the proximal stomach. Carcinoembryonic antigen (CEA) was noted to be 14.9 ng/ml (normal range: 0.0-3.0 ng/ml); cancer-antigen 15-3 (CA 15-3) was 210 U/ml (normal range: 0-31 U/ml); and CA 27.29 was 234.8 (normal range: 0-40 U/ml). The patient also had persistent anemia as well as non-infectious leukocytosis. Flow cytometry was performed and found to be negative for leukemia.

Given the high suspicion for a breast primary, a full thickness biopsy of the inframammary nodular lesions was attempted. Additionally, the patient underwent abdominal laparoscopy with peritoneal biopsies from the diaphragm, falciform ligament, and lower pelvic peritoneum. Interestingly, pathological results of the biopsies (chest wall and peritoneum) revealed an invasive lobular carcinoma. The patient was emotionally distraught and anxious after receiving the diagnosis. She received appropriate support and counseling from qualified providers.

The patient received one unit of packed red blood cells and symptoms improved. She was subsequently started on hormonal therapy with anastrozole, and later on the cyclin-dependent kinase (CDK) inhibitor, palbociclib.

In spite of extensive hematological work up, the patient continued to have leukocytosis. However, there was no bone pain or evidence of bone marrow involvement on PET scan. Bone marrow biopsy was then pursued. The patient was noted to have metastatic breast carcinoma with marrow replacement. She has since been scheduled for routine oncological follow up.

**Figure 4:**
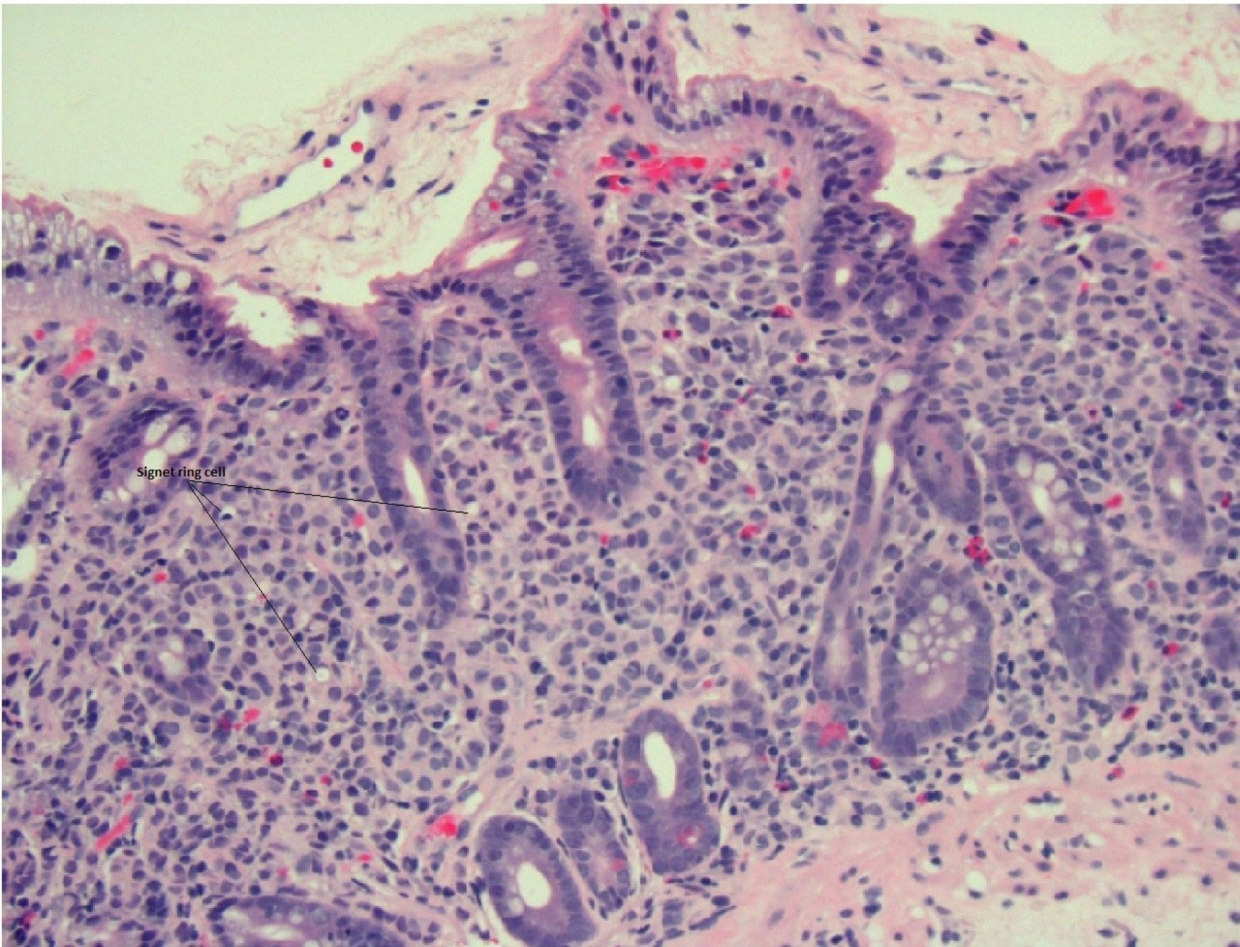
Duodenal biopsy revealing signet ring carcinoma; hematoxylin and eosin 20x

**Figure 5:**
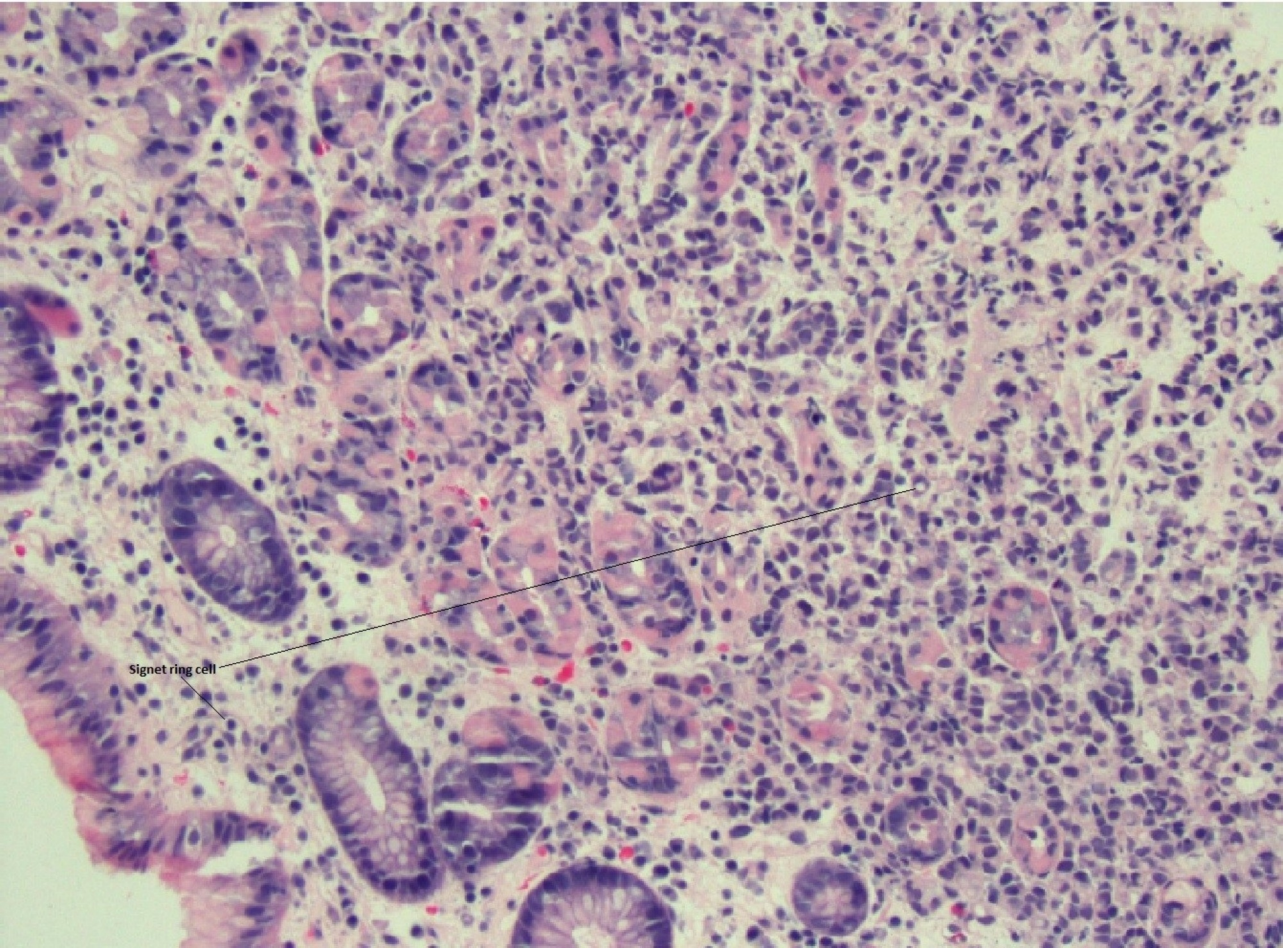
Gastric biopsy revealing signet ring carcinoma; hematoxylin and eosin 20x

**Figure 6:**
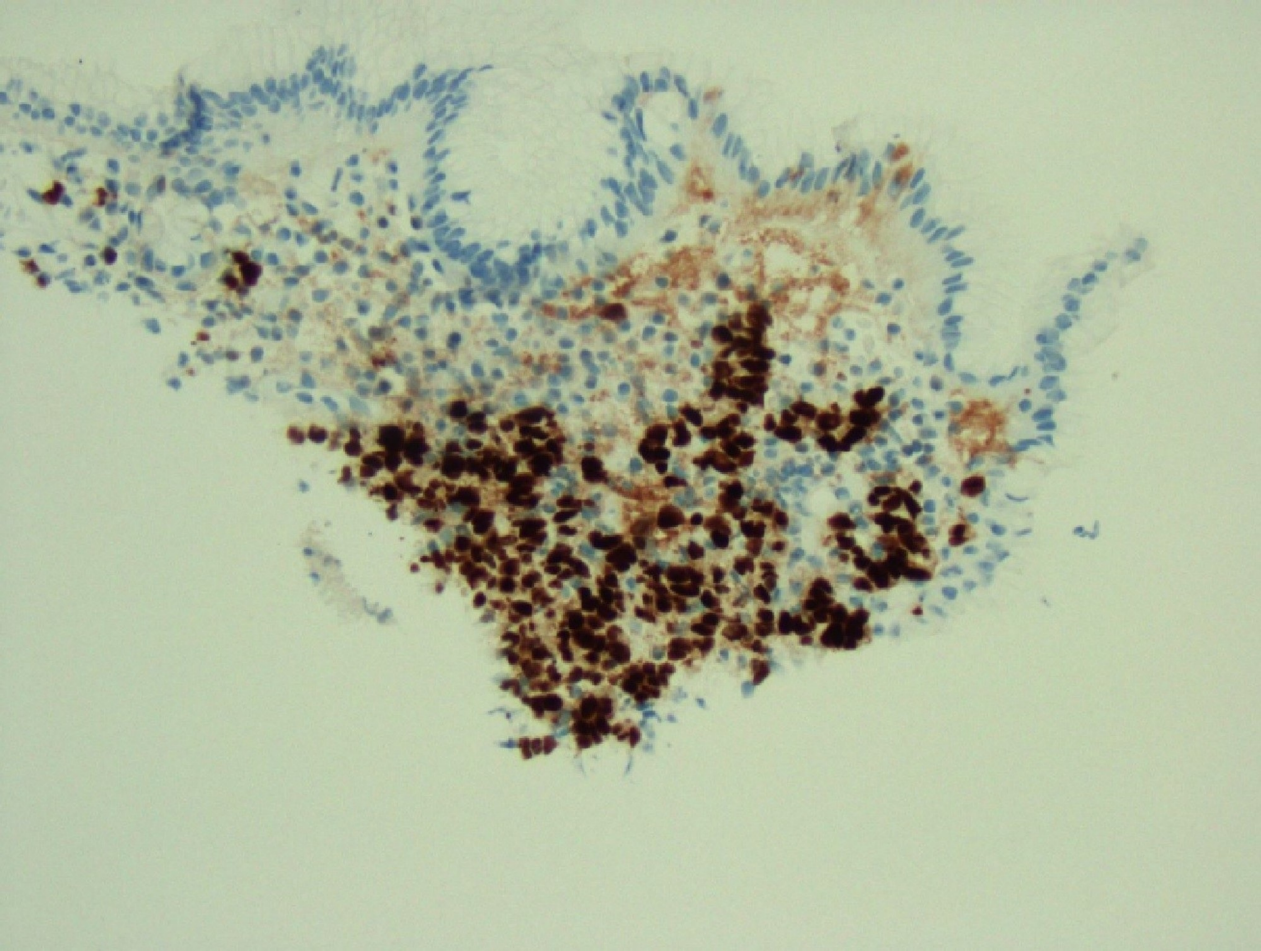
Gastric biopsy revealing signet ring carcinoma; immunohistochemical stain, estrogen receptor (ER)-positive 20x

**Figure 7:**
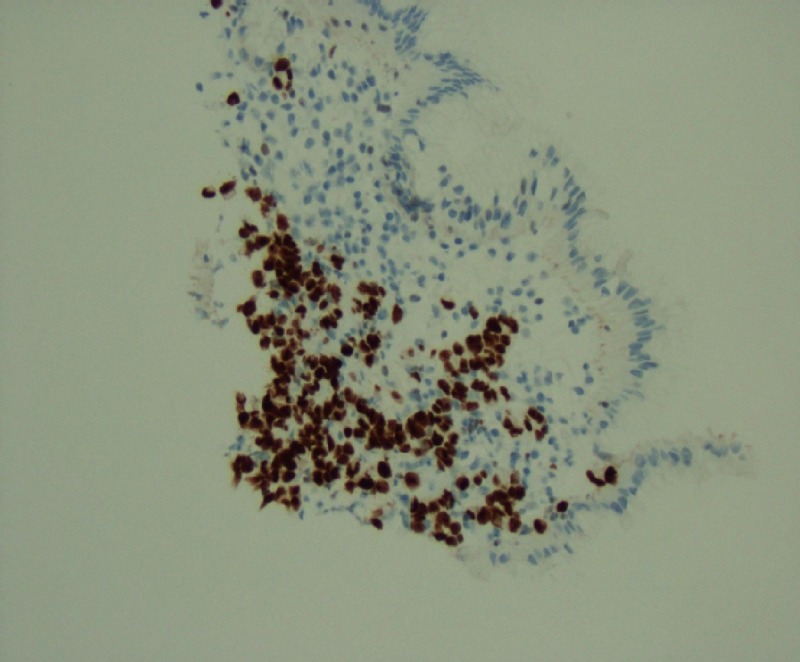
Gastric biopsy revealing signet ring carcinoma; immunohistochemical stain, GATA3-positive 20x

## Discussion

Invasive breast carcinoma most commonly metastasizes to the local and distant lymph nodes, bone, lung, liver, and brain. GI tract invasion as the first site of involvement is rare, and such metastases presenting as a sentinel event of severe anemia secondary to GI bleeding have seldom been described in the medical literature [5]. Morbidity associated with breast cancer metastases to the stomach has been estimated to be low [6]. However, it is conceivable that many cases are not identified due to the vague or non-specific constellation of symptoms that include indigestion, anorexia, and epigastric pain. Indeed, in many individuals, close examination via endoscopy is not pursued and the underlying etiology of symptoms is never definitively established. Our patient had a long history of non-steroidal anti-inflammatory drug (NSAID) use and acid reflux disease. As a result, providers initially attributed her upper GI bleeding to peptic ulcer disease from NSAID use. Furthermore, the endoscopic findings of erosive gastritis and duodenitis were consistent with frequent NSAID use.

Metastatic lesions from breast cancer to the GI tract usually develop several years after the primary tumor onset [5,7]; the reverse is true for GI manifestations preceding a diagnosis of breast cancer [8-9]. In our patient, suspicion for a primary breast cancer was exceedingly low, as she had undergone routine mammography per current guidelines with normal findings aside from the presence of fibroglandular breast tissue. Furthermore, MRI on admission was negative. Inasmuch as repeat imaging may have been appropriate for further screening if there was a high suspicion for breast cancer, there were no clinical features or family history to suggest the need for further workup. Indeed, the patient’s only significant risk factor was the use of topical estrogen for atrophic vaginitis.

In our patient, there were no gross breast masses noted on either examination or imaging. However, inframammary nodular lesions of the right chest wall were noted on physical examination. Full-thickness biopsy of the lesions was positive for lobular carcinoma. Importantly, the patient presented with the concomitant appearance of gastric and duodenal signet ring carcinoma with peritoneal involvement. Lobular carcinoma of the breast with metastasis to stomach sometimes demonstrates the appearance of linitis plastica, but this was absent on abdominal CT scan and laparoscopic examination. The unique nature of this case, therefore, lies in the synchronous appearance of the breast, gastric, and duodenal cancer with peritoneal involvement in the absence of a significant breast lesion on physical examination or imaging. Indeed, the only notable finding on breast examination was inframammary cobblestoning involving chest wall.

In individuals presenting with carcinoma of unknown primary, extensive histological and immunohistochemical evaluation is essential to establish a diagnosis; this was indeed required for our patient. The presence of ER/PR, GATA3, and mammaglobin and the absence of E-cadherin staining were highly suggestive of a breast primary, prompting further work up. Moreover, since 20% to 28% of gastric carcinomas express ER, thorough assessment of tumor markers may be important in guiding medical therapy.

It is important to note that lobular carcinoma of breast frequently takes the form of signet ring [10]. Whenever a primary gastric carcinoma such as gastric signet ring cell carcinoma is diagnosed, detailed work up should be performed to exclude invasive lobular carcinoma and optimize therapy.

It is critical not to overlook lobular carcinoma of breast because of its propensity for bilaterality. Indeed, bilateral lesions are estimated to be present in 46% to 96% of cases [11-12]. The diagnosis should be considered even in the absence of breast lesions or suspicious imaging findings, as early detection is essential to optimize patient outcomes.

The presentation of severe anemia from a GI bleed as a sentinel event of metastatic lobular carcinoma of the breast as sentinel event is rare. A high index of clinical suspicion, endoscopic biopsy, and thorough histological and immunohistochemical analyses are necessary to establish a diagnosis. Our hope is that this case increases awareness of the unusual presenting features of invasive lobular carcinoma of the breast that may be observed in individuals without obvious breast primary lesions.

## Conclusions

This case of metastatic lobular carcinoma of the breast could easily have been misdiagnosed as primary gastric carcinoma. Indeed, detailed clinical examination and histological analysis with immunohistochemistry were required to establish a definitive diagnosis. A thorough physical examination, as well as imaging and histopathologic studies, may be required to diagnose uncommon presentations of malignancies and select appropriate therapies.
